# Rapid non-destructive method to phenotype stomatal traits

**DOI:** 10.1186/s13007-023-01016-y

**Published:** 2023-03-31

**Authors:** Phetdalaphone Pathoumthong, Zhen Zhang, Stuart J. Roy, Abdeljalil El Habti

**Affiliations:** 1 School of Agriculture, Food and Wine, The University of Adelaide, Urrbrae, 5064 Australia; 2 The Waite Research Institute, Urrbrae, 5064 Australia; 3 Australian Institute for Machine Learning, The University of Adelaide, Adelaide, 5000 Australia; 4 Australian Research Council Industrial Transformation Training Centre for Future Crops Development, The University of Adelaide, Urrbrae, 5064 Australia

**Keywords:** Stomata, Phenotyping, Non-destructive, Handheld microscope, Machine learning

## Abstract

**Background:**

Stomata are tiny pores on the leaf surface that are central to gas exchange. Stomatal number, size and aperture are key determinants of plant transpiration and photosynthesis, and variation in these traits can affect plant growth and productivity. Current methods to screen for stomatal phenotypes are tedious and not high throughput. This impedes research on stomatal biology and hinders efforts to develop resilient crops with optimised stomatal patterning. We have developed a rapid non-destructive method to phenotype stomatal traits in three crop species: wheat, rice and tomato.

**Results:**

The method consists of two steps. The first is the non-destructive capture of images of the leaf surface from plants in their growing environment using a handheld microscope; a process that only takes a few seconds compared to minutes for other methods. The second is to analyse stomatal features using a machine learning model that automatically detects, counts and measures stomatal number, size and aperture. The accuracy of the machine learning model in detecting stomata ranged from 88 to 99%, depending on the species, with a high correlation between measures of number, size and aperture using the machine learning models and by measuring them manually. The rapid method was applied to quickly identify contrasting stomatal phenotypes.

**Conclusions:**

We developed a method that combines rapid non-destructive imaging of leaf surfaces with automated image analysis. The method provides accurate data on stomatal features while significantly reducing time for data acquisition and analysis. It can be readily used to phenotype stomata in large populations in the field and in controlled environments.

**Supplementary Information:**

The online version contains supplementary material available at 10.1186/s13007-023-01016-y.

## Background

Stomata are pores on the surface of leaves, stem and floral tissues of plants [1]. Stomata play an essential role in plant growth and plant response to abiotic and biotic stress. Approximately 98% of carbon dioxide (CO_2_) uptake and water loss from the plant occurs through stomatal apertures [2]. Stomata are dynamically regulated by environmental factors such as drought, heat, salinity, light, to name a few. When water supply is ample, stomata open to allow CO_2_ entry into the leaf for photosynthesis. Simultaneously, water is released to the atmosphere via transpiration [2, 3]. When water supply is limited, plants close stomata to prevent water loss, which also results in reduced CO_2_ assimilation and subsequently growth. The balance between CO_2_ assimilation and water loss is primarily important for adaptation to environmental cues without compromising growth [2, 3]. Stomata are also a gateway for pathogen entry into leaves and stomatal defence is a major physical defence mechanism in plant immunity [4, 5].

Stomatal gas exchange and its regulation are determined by stomatal morphology, density and sensitivity to the environment [6]. Plants show a range of stomatal sizes and shapes on the leaf epidermis depending on the plant species and variety [7]. Diversity in stomatal features allowed plants to adapt to a wide range of environments [8, 9]. Considering a changing climate, the flexible and dynamic nature of stomatal traits makes them primary targets for improving crop productivity and stability. Despite extensive research on stomatal biology, current knowledge is poorly translated into the context of field experiments and outputs for breeders. Conventional methods to phenotype stomata are time-consuming and costly, and do not allow screening large populations for beneficial stomatal traits. A commonly used method to investigate stomatal traits is a nail polish method (NP method) [10–15]. It consists of applying nail polish on the leaf surface to get a leaf imprint, waiting for the polish to dry, carefully peeling off the polish, examining the leaf imprint under a light microscope, taking and analysing images to determine stomatal traits. In addition to the length of time it takes to obtain a nail polish imprint, a common issue with this method is the unavoidable presence of air bubbles that interfere with stomata imaging [16], which can result in missing data and repeating the imprinting stage. Manual measurement of stomatal traits is time-consuming and inevitably introduces inconsistencies. Altogether, obtaining images of stomata from leaf imprints and analysing data is time-consuming, which limits the number of samples that can be analysed at any one time, making it impractical for phenotyping large mapping populations or diversity panels.

Recently, a number of methods have been published which address the issues with manual processing by including machine learning algorithms that automatically detect and analyse stomatal features [16–20]. While this improvement significantly accelerated image analysis and offers an effective substitute to manual analysis, these methods are limited by providing either information on stomatal number, size or aperture in one species in isolation; having data on all stomatal traits is desirable to have a comprehensive view on stomatal phenotype. In addition these faster image processing methods still depend on traditional time consuming approaches to obtain images, such as nail polish methods that require indirect leaf imaging from leaf imprints. Use of nail polish methods are impractical when working with large populations and having to take thousands of measurements from greenhouse or field grown plants, which require higher throughput image acquisition and analysis. To overcome indirect leaf imaging, approaches of directly taking images of leaves by bringing harvested plant material or whole plants to a microscope have been trialled [21–23]. Direct leaf imaging further accelerates stomatal phenotyping and demonstrates the potential of high-throughput stomata phenotyping tools in revealing novel insights on the genetic basis of stomata-related traits [24]. However, these methods are typically destructive or only provide partial information on stomata number and/or aperture.

**Fig. 1:**
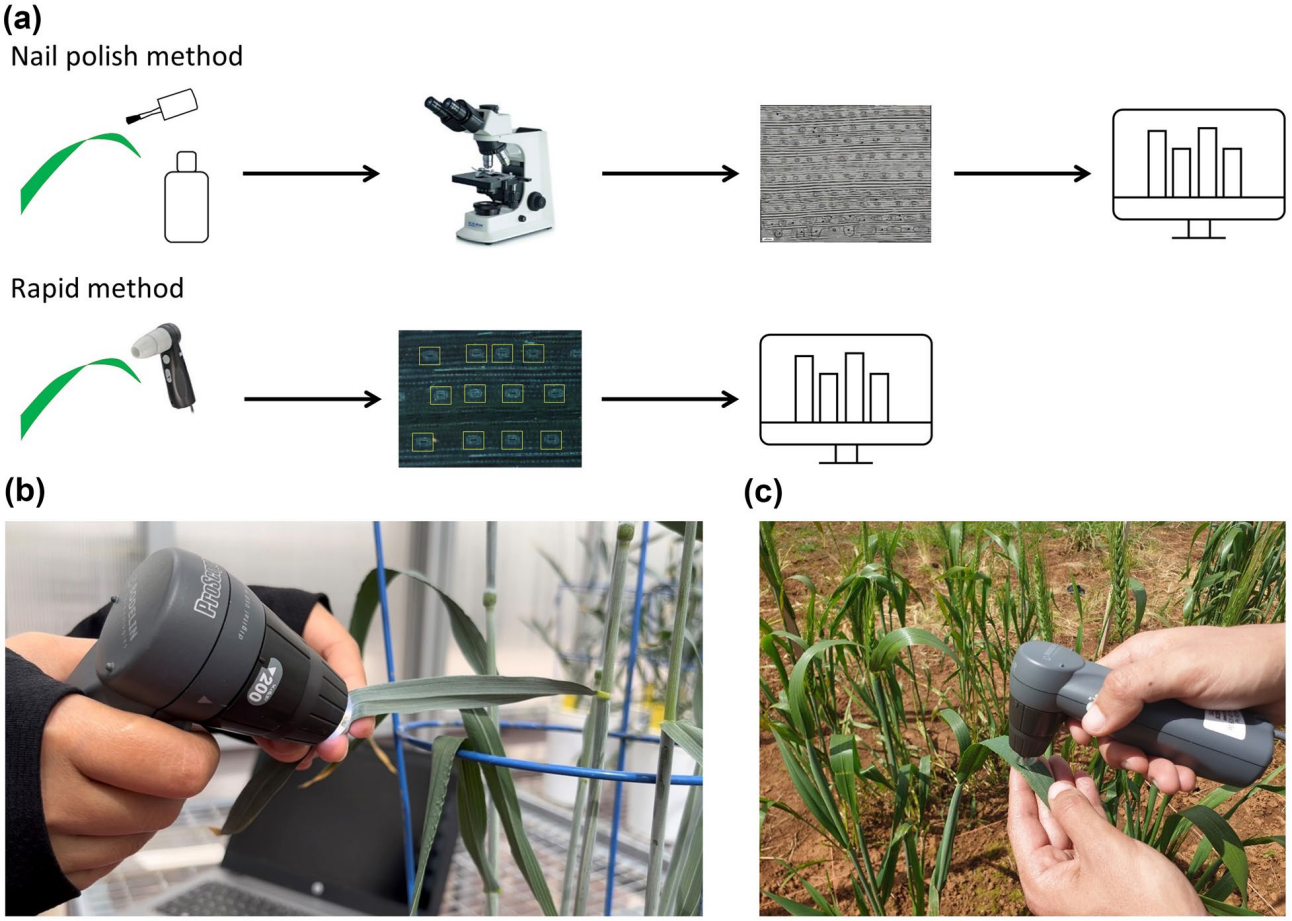
Overview of the rapid stomata phenotyping method (**a**) and experimental setup in controlled environment (**b**) and in the field (**c**)

Facing the need for a high-throughput method to phenotype stomatal traits in large populations and identify favourable stomatal traits, we developed a rapid non-destructive method for phenotyping stomata at large scale by combining a portable handheld microscope (HHM) for direct imaging of a leaf surface, with a machine learning model for automated stomata analysis (Fig. 1). This method was tested on three species: wheat, rice and tomato.

## Materials and methods

### Plant material

Three plant species were tested in this work: two monocotyledons (wheat—*Triticum. aestivum* cv. Cadenza and Gladius, and rice—*Oryza sativa* cv. R12) and one dicotyledons (tomato—*Solanum lycopersicum* cv. Sweetbite and Mighty Red). Wheat and tomato plants were grown in 20 cm pots containing UC Davis soil mix (50% peat and 50% sand) in a glasshouse located at the Waite campus (South Australia, 34°58′16.72″S latitude 138°38′23.17″E longitude) under natural photoperiod and 22 °C/15 °C day/night. Wheat was grown from June to October 2021 and tomato plants from July to November 2021. Rice was grown in 15 cm pots containing UC David soil mix in a glasshouse located at the Waite campus from September to December 2021 at 29 °C/21 °C day/night.

### Stomata imaging using the handheld microscope method

A handheld microscope (ProScope HR5, Bodelin, USA) was used to directly take images of plant leaves which were still attached to the plant. Three magnifying objectives were tested: 100 × lens (field of view: 2.87 × 2.17 mm; resolving power: 4 microns; pixel density: 1 mm = 198 pixels), 200 × (field of view: 1.36 × 1.03 mm; resolving power: 2 microns; pixel density: 1 mm = 415 pixels) and 400 × (field of view: 0.75 × 0.57 mm; resolving power: 1 micron; pixel density: 1 mm = 652 pixels). Images were captured using ProScope Capture v6.14 software.

### Stomata imaging using a nail polish method

For comparison with the HHM method, leaf imprints were collected by applying nail polish on the adaxial and abaxial leaf surface of 4-month-old wheat, 2-month-old rice and 3-month-old tomato. Nail polish (Insta-Dri Top Coat, Sally Hansen, USA) was applied on the leaf surface in a thin layer, and left to air dry for 5 min. Dry nail polish was peeled off using clear tape (Crystal clear Office tapes, Winc, Australia) and was placed on a microscope slide. Stomata on leaf imprints were observed using a light microscope (Nikon Ni-E compound microscope, Tokyo, Japan) connected to a DS-Ri1 colour cooler digital camera and a 40 × objective. Images of leaf imprints were taken using NIS-elements software (Nikon).

### Machine learning model training

#### Stomata detection model

Images taken with the HHM were used to train the machine learning model to detect stomata for each species and magnification used. Open-source software LabelImg [25] was used to annotate images by labelling a bounding box around individual stomata. The annotated images were uploaded to roboflow platform [26] for image augmentation. Images were random split into training, validation and testing sets (Additional file 1: Table S1). YOLOv5 algorithm [27] was used to train models for stomatal detection. For each species, a model was trained for 100 epochs with the default hyper-parameters in YoLOv5. The validation set was used to choose the best model evaluated with a test performance. Accuracy of stomata detection models was assessed with YOLOv5 using three parameters: precision, recall and F1 score. Precision is defined as

$$Precision = \frac{{\# \left( {True\;Positive\;Stomata} \right)}}{{\# \left( {All\;detected\;stomata} \right)}}.$$

Recall is defined as

$$Recall = \frac{{\# \left( {True\;Positive\;Stomata} \right)}}{{\# \left( {Total\;number\;of\;stomata} \right)}}.$$

F1 score is the harmonic mean of precision and recall and is defined as

$$F1 score = 2 \times \frac{Precision \times Recall}{{Precision + Recall}}.$$

#### Stomata measurement model

A similar protocol was followed to develop stomata measurement models. Data from the stomata detection models were used to extract individual stomata from the HHM images. Instance segmentation was done using Roboflow platform by manually outlining stomatal perimeter, including guard cells and subsidiary cells if applicable, and aperture of individual stomata. Individual stomata images were random split into training, validation and testing sets (Additional file 1: Table S2). Detectron2 platform [28] was used to train the model for measurement of stomatal area and aperture. For each species, a model was trained for 300 epochs with learning rate of 0.00025. The validation set was used to choose the best model evaluated with a test performance.

#### Comparative analysis of automated vs manual measurements

Data on stomatal traits measured using the developed machine learning models were compared with data measured manually on 100 images. The number of stomata was counted visually. Stomatal size and aperture were measured manually using Fiji [28] by outlining stomatal perimeter and aperture.

## Results

### Optimal magnification for each species

**Fig. 2:**
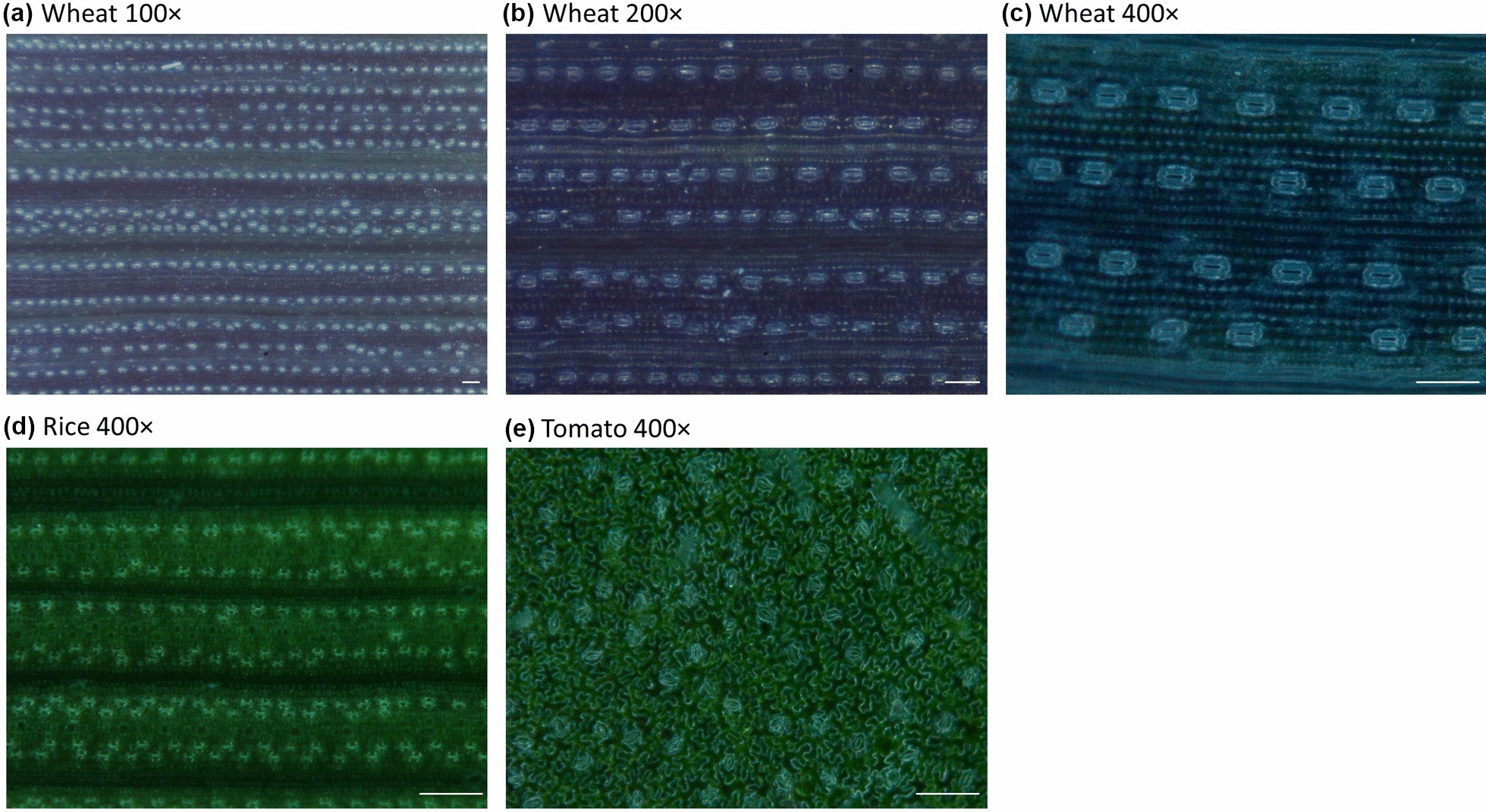
Handheld microscope images of wheat leaves with 100 × (**a**), 200 × (**b**) and 400 × (**c**) magnification, rice (**d**) and tomato leaves (**e**) with 400 × magnification. Scale bar = 100 μm

To assess the suitability of each magnification in providing accurate data on stomatal traits, three magnifying objectives were tested: 100 × , 200 × and 400 × . The suitability of a magnification in quantifying stomatal traits depended on the size of stomata in each species (Fig. 2). In wheat, the large field of view of the 100 × magnification allowed the counting of stomata over a large area, but the lower resolution did not allow accurate measurement of stomata size (Fig. 2a). The intermediate specifications of 200 × magnification enabled both counting the number of stomata in a smaller area on the wheat leaf and could be used to determine stomatal size (Fig. 2b). The high resolution of 400 × magnification was optimal for determining stomatal size and aperture in wheat (Fig. 2c). Stomata in rice and tomato are significantly smaller than stomata in wheat and therefore only the 400 × magnification could be used to determine the number and size of stomata (Fig. 2 d-e).

### Comparison between nail polish and handheld microscope images

**Fig. 3:**
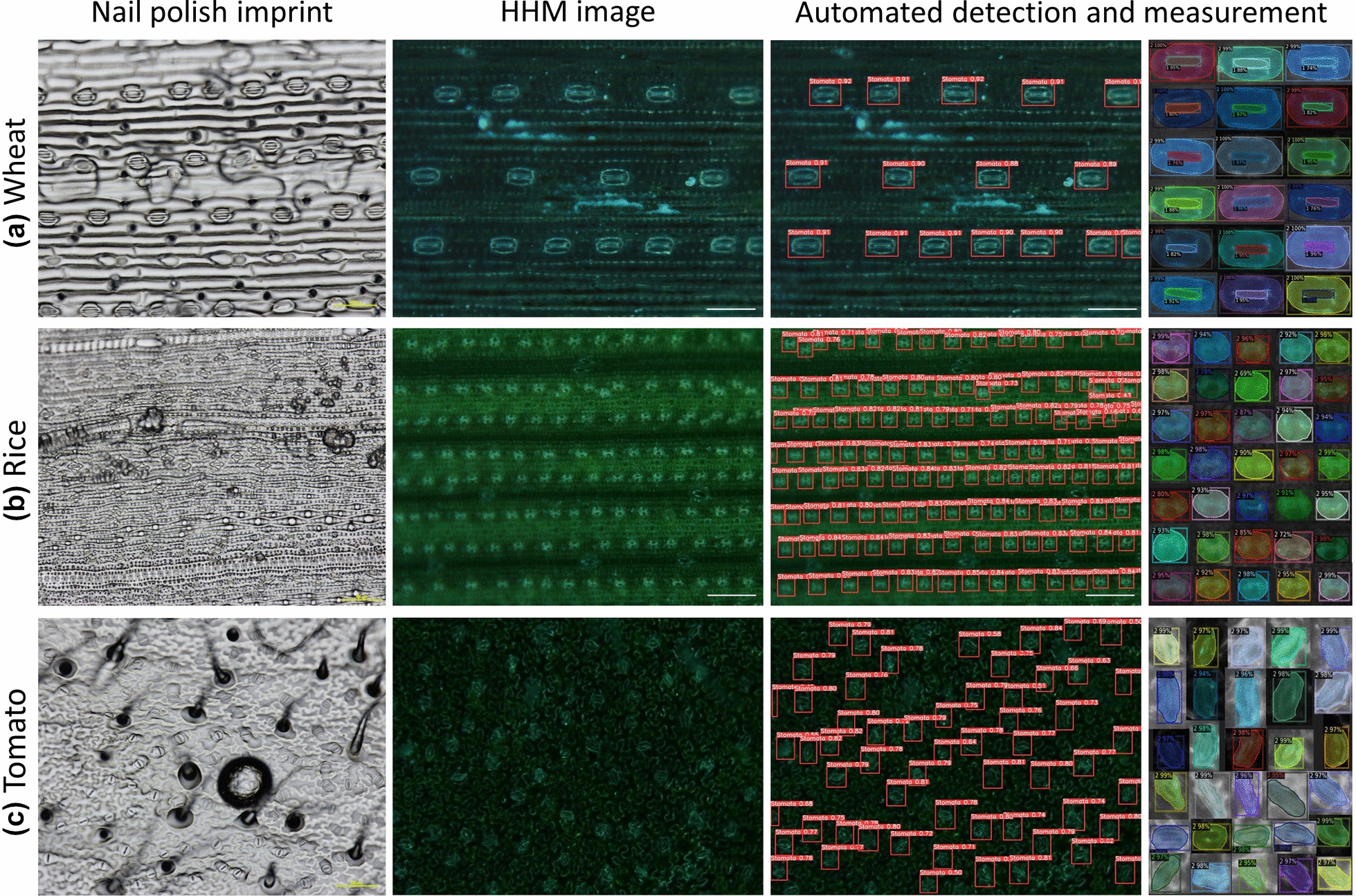
Qualitative comparison between images from nail polish imprint images and handheld microscope images of wheat (**a**), rice (**b**) and tomato leaves (**c**), and automated detection and annotation of stomata and aperture. Scale bar = 100 μm

The HHM provided good quality images faster than the nail polish method. Although leaf imprint images taken with a light microscope were generally at higher resolution than the HHM images, air bubbles were frequently present in imprint samples (Fig. 3). Handheld microscope images of wheat leaves using 400 × magnification were at high resolution given the large size of stomata in this species and allowed automated measurement of number of stomata, stomatal size and aperture (Fig. 3a). In rice and tomato and leaves, images taken with the HHM were at a lower resolution compared to light microscope images given the smaller size of stomata in these species, but stomata were distinct enough to allow for automated detection of stomata and measurement of stomata number and size (Fig. 3b-c). Rice leaf imprints were uneven and required taking two images at different focus to visualise all stomata present on the leaf. The HHM allowed the observation of all rice stomata in one image (Fig. 3b). Given their smaller size, stomata in rice leaves could only be observed using the 400 × magnification but image quality was still suitable for machine learning. In tomato, leaf imprint images were as clear as images taken with the HHM and stomata could be observed with a 400 × magnification (Fig. 3c).

### Data accuracy of detection and measurement models

The machine learning models were highly accurate in detecting stomata in wheat, rice and tomato. Model accuracy depended on the species and the magnification used (Table 1, Additional file 1: Fig. S1). In wheat, model accuracy was highest with 400 × images (F1 = 0.99) compared to 200 × (F1 = 0.91) and 100 × (F1 = 0.89), as stomata are more distinct at higher magnification. When comparing across all species using the 400 × magnification, stomatal detection in wheat images had the highest accuracy compared to rice and tomato (F1 = 0.99, 0.90 and 0.87, respectively).

**Table 1:** Detection models’ statistics

Species	Magnification	Precision	Recall	F1 score
Wheat	400 ×	0.993	0.998	0.995
Wheat	200 ×	0.898	0.917	0.907
Wheat	100 ×	0.977	0.815	0.889
Rice	400 ×	0.868	0.926	0.896
Tomato	400 ×	0.836	0.913	0.873

**Fig. 4:**
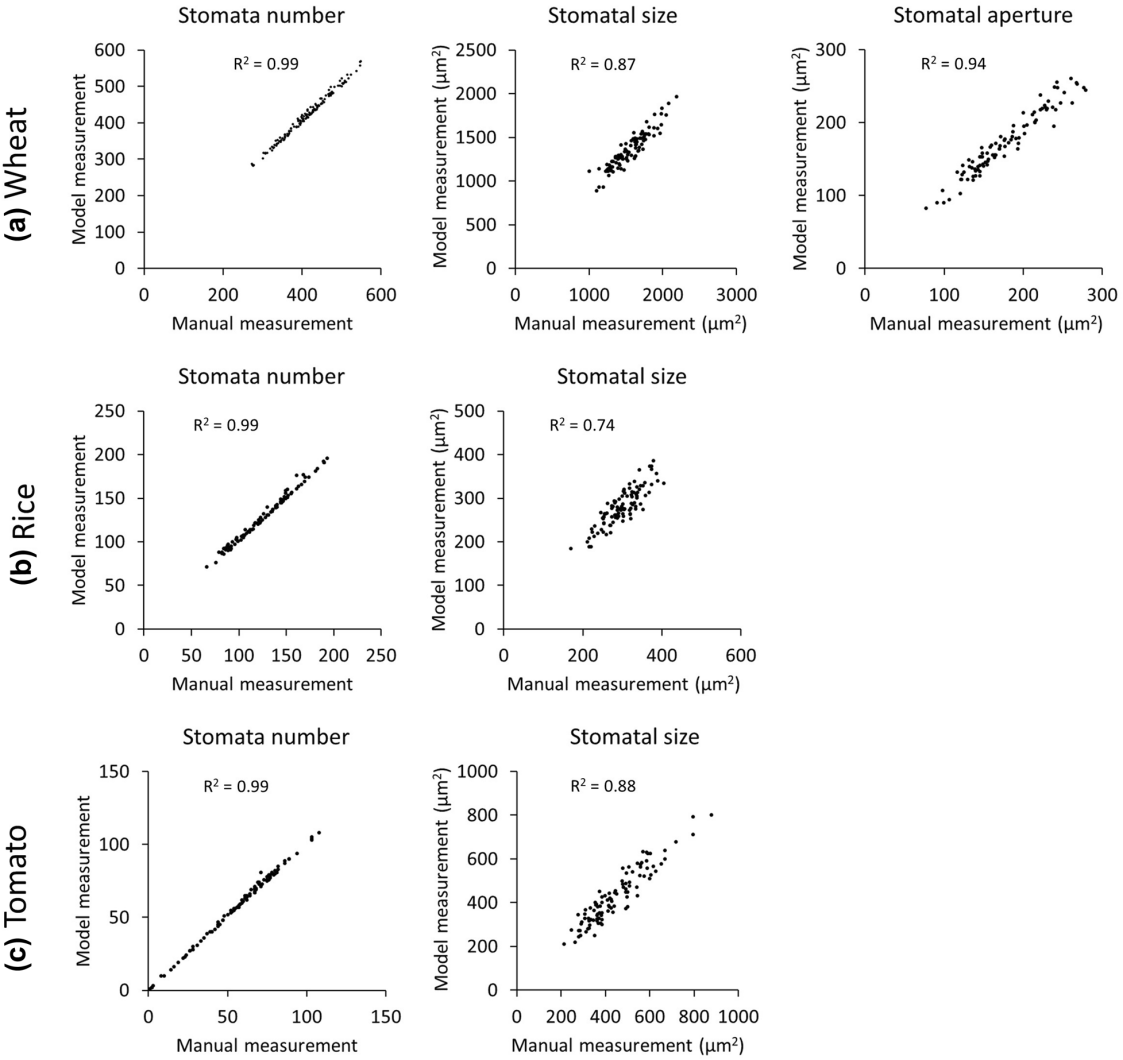
Scatterplots of stomatal traits comparing data measured by machine learning models with manual measurements of 100 images of wheat (**a**), rice (**b**) and tomato leaves (**c**). R^2^ is the coefficient of determination of a linear regression between computed and manual counts

**Fig. 5:**
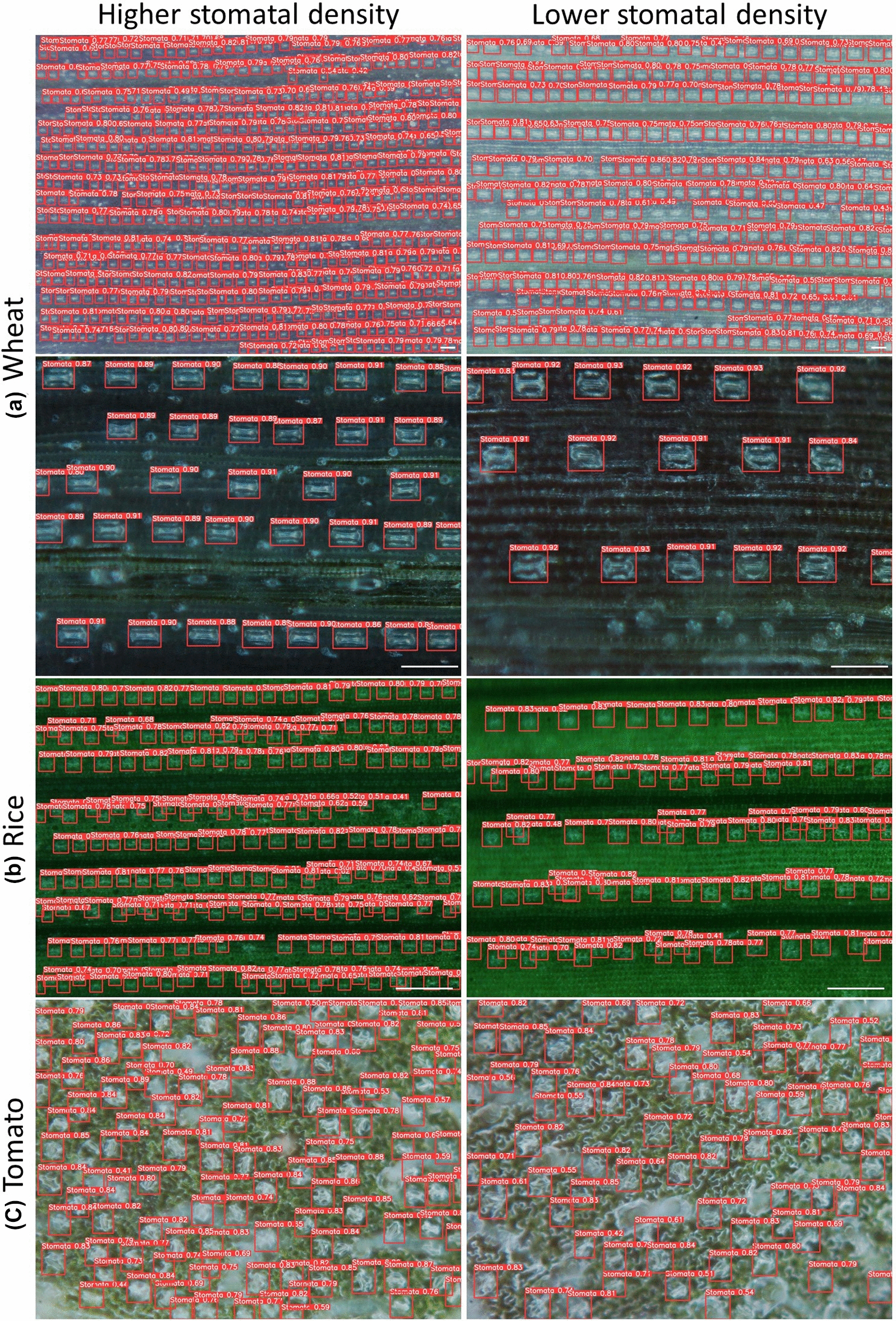
Rapid identification of contrasting stomatal phenotypes: high stomatal density (left) and low stomatal density (right) in wheat (**a**), rice (**b**) and tomato (**c**). Scale bar = 100 μm

The developed models were able to detect nearly all of the stomata in an image and to measure stomatal size and aperture (Fig. 4). In wheat, data from machine learning models were highly correlated with manual measurements for stomata number (R^2^ = 0.99), stomatal size (R^2^ = 0.87) and stomatal aperture (R^2^ = 0.94) (Fig. 4a). In rice, data from machine learning models were highly correlated with manual measurements for stomata number (R^2^ = 0.99). The correlation was lower for stomatal size (R^2^ = 0.74) (Fig. 4b). In tomato, data from machine learning models were highly correlated with manual measurements for stomata number (R^2^ = 0.99) and stomatal size (R^2^ = 0.88) (Fig. 4c). The accuracy of detection and measurement models in wheat, rice and tomato allowed rapid identification of contrasting stomatal number and size; high stomatal density with smaller stomata, and low stomatal density with larger stomata (Fig. 5).

## Discussion

Research on crop adaptation to biotic and abiotic stresses involves phenotyping large populations to identify germplasm with desirable traits. This requires non-destructive, high-throughput phenotyping tools that accurately measure traits of interest [29]. The current low throughput methodology for studying stomatal physiology limits its use in forward genetics screens. Given the central role of stomata in plant physiology, a rapid method was needed to capture the diversity in stomatal traits in plants [24]. One of the principles of non-destructive phenotyping is to bring the instrument to the plant, rather than the plant to the instrument. Portable handheld microscopes are therefore convenient tools for non-destructive imaging of the leaf surface. The method described here overcomes the tedious image acquisition process by using a HHM to acquire images directly from the leaf of plants growing in natural conditions. The HHM provided clear images of wheat, rice and tomato leaf surface in a few seconds, rather than minutes as is the case with the NP method. Images taken with a HHM can be viewed immediately, and alternative images of the same leaf can be taken if image quality is not satisfactory.

In rice and tomato, the small size of stomata only allowed the use of 400 × lens, the highest magnification currently available. This magnification provided satisfactory images to count and measure stomata in the three species. Wheat has larger stomata that were visible with 100 × , 200 × and 400 × magnifications. The 100 × lens offers a wider field of view and therefore provides a more representative view on stomata number. The 400 × lens offers higher resolution of stomata and allows accurate quantification of stomatal size and aperture. The high resolution of the 400 × lens makes it possible to record videos of the leaf surface in wheat and to observe and quantify the dynamic changes of stomatal aperture in response to environment, a technique successfully tested in wheat [21]. The 200 × lens offers a satisfactory compromise between field of view and resolution and can be used to phenotype all stomatal traits in wheat using the same image, which further reduces the time of image acquisition.

This method described here combines the advantages of a portable HHM (rapid, non-destructive acquisition of stomata from leaves on plants in their growing environment), with automated accurate analysis of stomatal features, thus making stomata phenotyping significantly faster than conventional methods. The method was successfully applied to identify contrasting stomatal phenotypes, which is the ultimate purpose of a stomata phenotyping tool. The rapid method is affordable and can be readily used since it does not require specific skills in computer science or programming. The stomata image analysis pipeline is publicly available for each species [30]. HHM images can be analysed immediately using the machine learning model.

## Conclusion

Our stomatal phenotyping method provides a rapid, non-destructive tool to determine stomata number, size and aperture if applicable. The experimental setup is portable and allows stomata phenotyping at a large scale, in controlled environments and in the field. This screening tool will accelerate research in stomatal biology in the context of increasing biotic and abiotic pressure on crop production. The method is versatile and can be further adapted to more species using the same HHM and image analysis pipeline.

## Supplementary Information

**Additional file 1**: **Fig. S1**. Confidence curves of detection models of wheat with 100× (a), 200× (b) and 400× (c) magnification, rice (d), and tomato (e) with 400× magnification. **Table S1. **Number of images used to develop stomata detection models. **Table S2. **Number of images used to develop stomata measurement models.

## Data Availability

The datasets generated and analysed during the current study are available in github repository “rapidmethodstomata”, https://github.com/rapidmethodstomata/rapidmethodstomata.
