# Supplementary material for: Rapid non-destructive method to phenotype stomatal traits
Source: Plant Methods. 2023 Mar 31;19:36. doi: 10.1186/s13007-023-01016-y (PMC10064510; doi:10.1186/s13007-023-01016-y)
Supplement: Supplementary file 1 — Additional file 1: Fig. S1. Confidence curves of detection models of wheat with 100× (a), 200× (b) and 400× (c) magnification, rice (d), and tomato (e) with 400× magnification. Table S1. Number of images used to develop stomata detection models. Table S2. Number of images used to develop stomata measurement models. [file 13007_2023_1016_MOESM1_ESM.docx]

**Additional file 1**


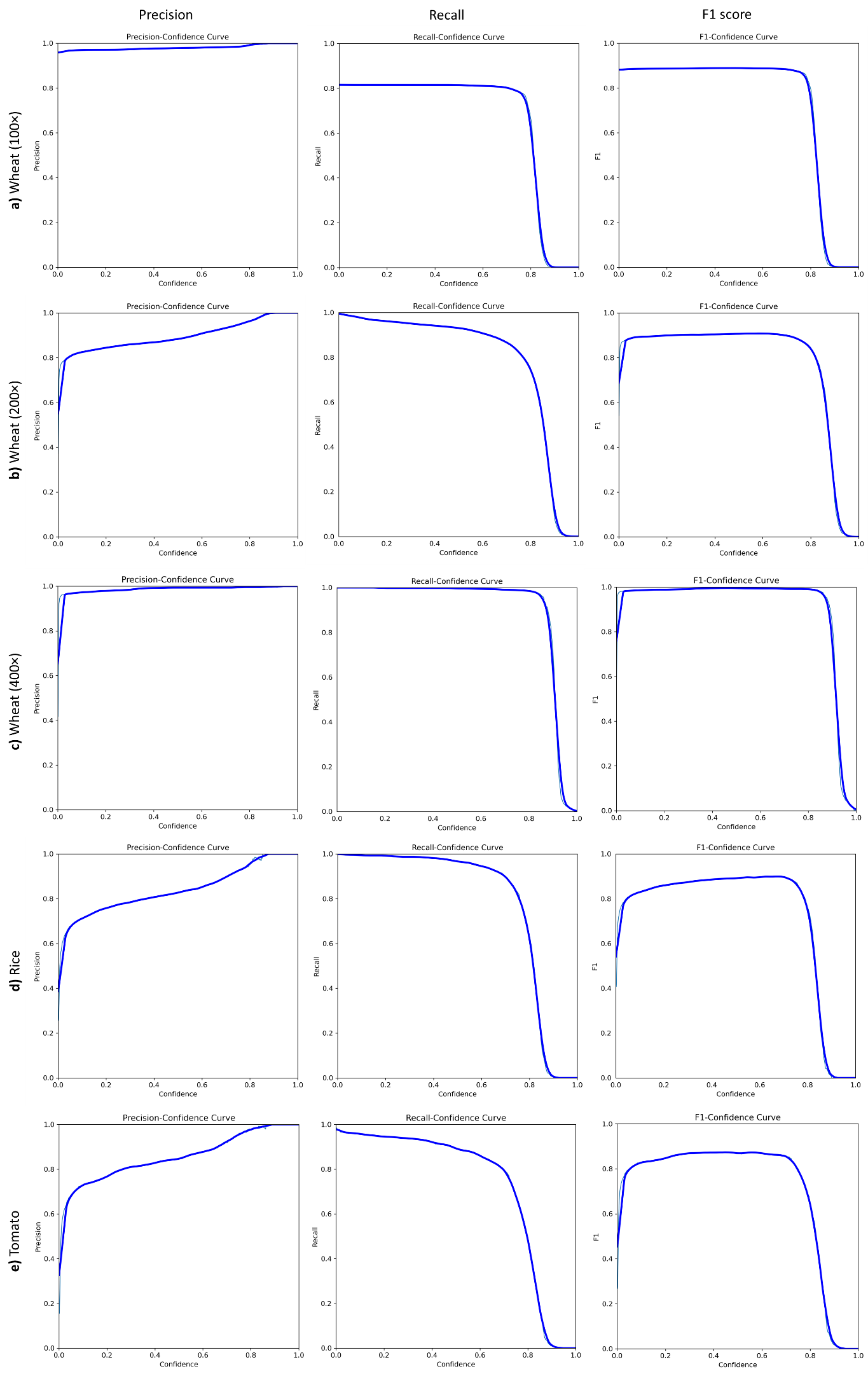


**Fig. S1: Confidence curves of detection models of wheat** with 100× (a), 200× (b) and 400× (c) magnification, rice (d), and tomato (e) with 400× magnification.

|  | | **Number of images** | | |
| --- | --- | --- | --- | --- |
| **Species** | **Magnification** | **Training set** | **Validation set** | **Testing set** |
| Wheat | 400× | 417 | 40 | 20 |
| Wheat | 200× | 486 | 46 | 23 |
| Wheat | 100× | 450 | 42 | 21 |
| Rice | 400× | 183 | 18 | 9 |
| Tomato | 400× | 216 | 20 | 10 |

**Table S1:** Number of images used to develop stomata detection models.

|  | | **Number of images** | | |
| --- | --- | --- | --- | --- |
| **Species** | **Magnification** | **Training set** | **Validation set** | **Testing set** |
| Wheat | 400× | 297 | 83 | 44 |
| Rice | 400× | 280 | 80 | 40 |
| Tomato | 400× | 295 | 85 | 43 |

**Table S2:** Number of images used to develop stomata measurement models.
